# Use of fungal alpha amylase and ascorbic acid in the optimisation of grain amaranth–wheat flour blended bread

**DOI:** 10.29219/fnr.v62.1341

**Published:** 2018-09-28

**Authors:** Ruth J. Kamoto, William Kasapila, Tinna A. Ng’ong’ola-Manani

**Affiliations:** Department of Food Science and Technology, Lilongwe University of Agriculture and Natural Resources (LUANAR), Lilongwe, Malawi

**Keywords:** improver, blending, fermentation, oven spring

## Abstract

Grain amaranth–wheat flour bread was optimised using ascorbic acid (0.03% dry weight basis) and fungal α-amylase (10 ppm) to investigate their effects on sensory properties of the breads. Six formulations were used in the ratios of 5:95, 10:90, 15:85, 20:80 and 25:75 grain amaranth to wheat flour compositions, while the control bread had 100% wheat flour. Consumer acceptability and preference ranking tests were conducted to describe and evaluate preference and acceptability of the breads. Analysis of consumer acceptability data revealed that there were significant differences (*p* < 0.05) for all the samples in all the attributes tested. Overall consumer acceptability results showed no difference at 25% for all improvers. Principal component analysis for descriptive tests performed by a trained panel demonstrated variations among the breads in graininess, elasticity, crumb colour, stickiness and crumb moistness regardless of the improvers used. The study concludes that using improvers to optimise grain amaranth–wheat flour bread can help improve both the nutritional and organoleptic properties of bread.

Amaranth, a plant that grows wildly in many countries of the world, has been underutilised for years. A 1977 article by Jean L. Marx in the journal *Science* has already described amaranth as ‘the crop of the future (1)’.

Amaranth is nutritious, stuffed with vitamins, folic acid (vitamin B9), minerals and protein. The plant is edible from tender stems through leaves, flowers and grains. Cooked leaves can be used variously as simple green side dishes, in quiches, pies, toppings and soups. Amaranth grains can be boiled and used to make candies, herbs and pressed seed oil with commercial uses besides toasting much like popcorn and mixed with honey, molasses and chocolate.

In Malawi and other countries in Africa, amaranth leaves are merely eaten as side dishes or relish for maize, rice and banana meals. The grains are left on the plant and consumed by birds or blown away by wind to far-flung areas from people’s homes, further making it wild.

Culturally, people in Africa consider this grain inedible, and the problem is compounded by lack of recipes, processing techniques and knowledge of how to incorporate it into the current diets. According to the Educational Concerns for Hunger Organization (ECHO), amaranth contains anti-nutritional factors, including oxalates, nitrates, saponins and phenolic compounds that are reduced in content and effect by cooking (2).

Yet, as befits its weedy life history, amaranth grains grow rapidly and, in most of the wild species, their large seed heads can weigh up to 1 kg and contain a half-million small seeds (3). The grains are very small, about 1 mm in diameter, and difficult to harvest into meaningful volumes for commercial use.

Recently, there has been growing interest in the production of grain amaranth due to its potential in improving nutrition, food security and rural incomes. For example, from the late 1990s, the Indigenous Vegetables Research Project at Bunda College of Agriculture in Malawi has been breeding amaranth predominantly for its grains. The improved grains are golden in colour, lenticular and relatively larger (up to 1.7 mm diameter) than those from wild varieties. A number of humanitarian organisations have been distributing seeds for these varieties to rural households for them to grow in home gardens as part of nutrition interventions.

The United Nations Children’s Fund and Food and Agriculture Organization (4) have already identified Malawi as one of the 34 countries (22 from Africa) that account for 90% of the global burden of stunting, with 37 in every 100 children being short for their ages, and called for innovative techniques to improve the nutritional content of foods.

This study used flour from improved grain amaranth as an ingredient in wheat bread to promote its adoption and utilisation in diets.

Breads are an important staple food and suitable for nutritional improvement.

Amaranth grain is high in linoleic acid, protein and lysine, an amino acid found in low quantities in many grains (5), including maize, sorghum, rice and bananas. Amaranth grain is deficient in essential amino acids such as leucine and threonine – both of which are present in wheat germ (5). These variations and complementarities that can be achieved make blending of amaranth and wheat flours all the more important.

Amaranth grain does not contain gluten, which makes it a viable grain for people with gluten intolerance.

However, blending amaranth grain flour with wheat affects the rheological properties of wheat, which, in turn, limits the baking characteristics and quality of the final bread. This study blended best proportions of grain amaranth and wheat flour besides using fungal α-amylase (FA) and ascorbic acid (AA) as improvers to produce good quality and nutritious bread.

Gluten is a composite of storage proteins termed prolamins and glutelins and stored together with starch in the endosperm of various cereal grains, in particular wheat, barley, rye and oats (6, 7). It gives elasticity to dough, helping it rise and keep its shape, and often gives the final product a chewy texture. Gluten is prepared from flour by kneading the flour under water, agglomerating the gluten into an elastic network, a dough, and then washing out the starch.

The results of this study can benefit nutrition practitioners, researchers, rural communities and the food industry to explore ways of commercialising the grains from amaranth and diversify selection of breads.

## Materials and methods

### Sample acquisition

Grain amaranth (*Amaranthus hypochondriacus*) was purchased from the horticulture department at Lilongwe University of Agriculture and Natural Resources (LUANAR) and stored in a sack to allow for aeration. Wheat flour, manufactured by Capital Foods, Sunfoil vegetable cooking oil (sunflower oil), Anchor instant yeast, table salt and white sugar were purchased from local shops in the country. Ascorbic acid was supplied by Lab Supplies, and fungal α-amylase enzyme (EN01 Bakezyme P500) manufactured by Lallemand, Montreal, Canada, was purchased from Anchor Yeast, South Africa.

Grain amaranth was sorted to remove physical objects like stones, washed several times to remove dust with clean tap water and dried in the food dryer in the Foods Laboratory at Bunda campus until all the grains were fully dried. It was then ground into flour using a blender (high horse power XTY-767, Taiwan Technical, Taiwan, China) sieved (0.5 mm) several times until almost all the grains were fine. The flour was kept at room temperature in an airtight plastic container until use.

### The bread making process

**Fig. 1:**
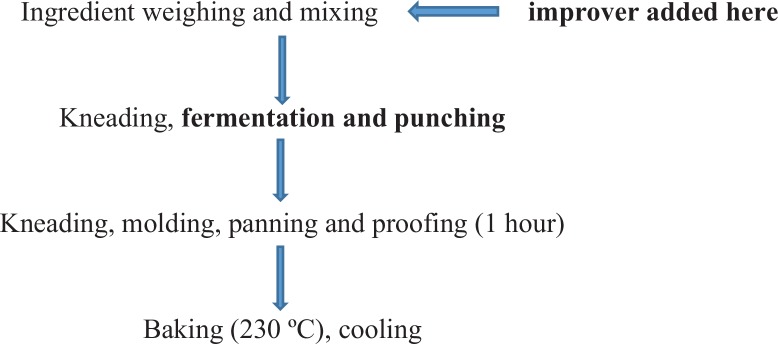
Flow diagram for bread production.

The bread making recipe was adopted from Tang et al. (8), with a few alterations due to the addition of improvers. The basic formula was 400 g flour, 6 g yeast, 30 g white sugar, 4 g salt, 32 g vegetable cooking oil and 230 g water. Grain amaranth flour substitution levels were from 5 to 25%. Ascorbic acid 0.03% (dry weight basis) was used as an improver, while 10 ppm FA was incorporated in each formulation following the manufacturer’s instructions. A series of trials were done to standardise the recipe based on the preliminary sensory tests carried out. Bread making processes proposed by Igbabul, Num and Amove (9) were followed (see Fig. 1). The processes in bold were included for maximum manipulation of dough.

The ingredients (wheat flour, grain amaranth flour, sugar, salt, yeast and improvers) were blended for 5 min in a mixer (KitchenAid, Model 5K5SS, MI, USA) and mixed with water and vegetable oil to develop dough. The dough was turned onto a flat clean surface and kneaded manually for 8 min. After kneading, the dough was allowed to rest for 20 min for the first fermentation at 25°C and 72% relative humidity (indoor thermometer and hygrometer, TH101, Mingle, China) using Gallenkamp Hotbox oven size 2. Punching was then done to further develop gluten and redistribute the nutrient supply for yeast, resulting in increased rates of fermentation and gas production (10). The dough was kneaded again for 5 min after which it was divided, moulded and placed in baking pans (21×10×7 cm^3^). Proofing was done for 40 min at 38 °C and 79% relative humidity, and then baking was done at 230 °C for 20–25 min. Breads were removed from the pans and cooled for 1 h and stored at room temperature for 16 h before sensory evaluation.

## Ethical consideration

This study was approved by the Ethics Committee of the Lilongwe University of Agriculture and Natural Resources. Actual participation in the study was based on full consent from the students and the headmistress. The researcher informed the students that participation was voluntary and that they had the right to withdraw from the study anytime if they wished so. All the responses given were kept confidential and the forms used for data collection were not shared with anyone outside the team.

## Sensory evaluation

### Consumer acceptability test

**Fig. 2:**
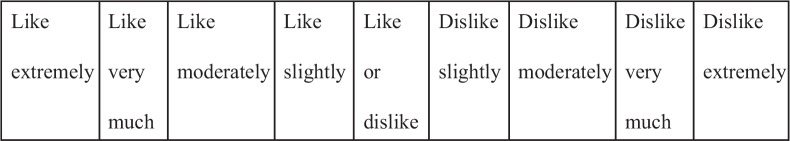
Representation of a 9-point hedonic scale.

Test breads were evaluated by 80 day scholars, 39 females and 41 males aged between 14 and 18 years, from Mkwichi Secondary School. The criterion for inclusion was that the testers must make an informed decision and give consent to participate in the study. The testers were also required to be familiar with sensory qualities of bread to be included in the study. A 9-point hedonic scale (Fig. 2), also known as the degree–of-liking scale, was used by the testers to indicate the extent to which they liked or disliked each bread sample. The scale ranged from 1 ‘like extremely’ to 9 as ‘dislike extremely’.

The study was conducted in two spacious, quiet and well-lit classrooms accommodating 20 testers each. The tests were conducted in three sessions. Samples of 5, 10, 15, 20 and 25% amaranth and 100% wheat flour with ascorbic acid as an improver were presented in the first session. Bread samples improved with FA and a combination of ascorbic acid and fungal α amylase (AFA) were tasted in the second and third sessions respectively. All six samples were served at room temperature and presented all at once with a questionnaire in their respective sessions. Consumers were asked to rate each sample on appearance, taste at first bite, aroma, roughness or graininess, aftertaste and overall acceptability. The tests were done at mid-morning when the testers were neither too full nor too hungry. The samples were presented at room temperature in small white plates and were coded and presented randomly using 3 digits. The testers were instructed to taste the samples from left to right in the given order, and no re-tasting was allowed. Clean tap water was provided in disposable cups for palate cleansing before the testers moved on to the next sample (11, 12).

### Preference ranking tests

**Fig. 3:**
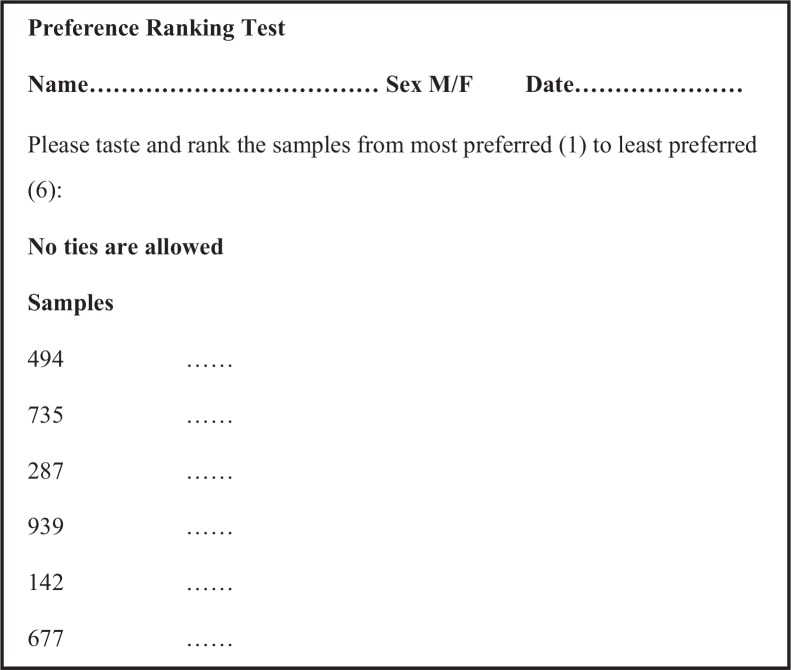
Preference test questionnaire.

Preference ranking tests were conducted concurrently with acceptability tests in a single session for each batch. After tasting all the six samples in consumer acceptability test, the testers were instructed to rank them from 1 to 6, where 1 referred to the ‘most preferred’ and 6 the ‘least preferred’ sample. No ties were allowed in this regard (Fig. 3). The consumers were given a gift as a token of appreciation at the end of each session.

### Descriptive test

Descriptive analysis was conducted by a trained panel of students and staff members at LUANAR in which they described the sensory properties of grain amaranth–wheat blended bread optimised by ascorbic acid, FA and a mixture of the two.

## Screening and selection of study subjects

Food science and technology students responded to an invitation made through notice boards for a screening exercise. A triangle test was used to screen panelists who volunteered to participate in the study. In this regard, the panelists were presented with three sets of samples to assess their accuracy in determining sweetness, saltiness and bitterness. Each set of samples for one panelist had one reference sample with similar formulation to one of the two remaining samples on the tray. For sweetness, besides the reference sample, the two samples had 0.1 and 0.15 molar sugar solutions, while the second set was made of 0.1 and 0.15 molar salt solutions. The third set was for the bitterness test, and two of the three samples contained 0.05 and 0.1 molar of quinine. Finally, 10 panelists were selected based on their ability to discriminate small test differences in terms of sweetness, saltiness and bitterness in the exercise.

## Training of panelists

The researcher briefed the selected panellists about the study and trained them at LUANAR for 4 weeks, with three 1-h sessions per week. The training started with language development where the panellists generated descriptors to be used for the sensory attributes of breads. Proper definitions for the descriptors were generated by the panellists after reaching a consensus. A total of 17 descriptors were generated under appearance, aroma, taste and texture items. Standard references were used for some of the descriptors, and a 15-point line scale was used in which 1 was rated ‘none or weakest’ and 15 was described as ‘extremely intense’ for a particular attribute. The descriptive test was conducted on fresh samples every other day in triplicate. Serving of the samples was done in small Ziploc bags to prevent dryness and sequential effect on some of the attributes, for example, crispiness. Crust and crumb colour, earthy smell, yeasty smell, sweetness, alveolus size and regularity were some of the attributes examined. An assessment of panel consistency was done using panel check V 1.4.2 (13). The assessors who were not consistent were provided with further training.

## Determination of physical characteristics of bread

Oven spring was estimated from the difference in height of the dough before and after baking. Loaf weight was measured 30 min after the loaves were removed from the oven using a laboratory scale, and the readings were recorded in grams (14). Loaf volume was measured using the rapeseed displacement method (15). Thus, a box of fixed dimensions (15×18×30 cm^3^) was placed in a tray, half filled with sorghum grains, shaken vigorously four times, and then overfilled slightly to allow the overspill fall into the tray. The box was shaken again twice before a straight edge was used to press across the top of the box once to give a level surface. The grains were decanted from the box into a receptacle and weighed. The procedure was repeated three times, and the mean value for grain weight was noted in grams.

A weighed loaf was placed in the box, and weighed grains were used to fill the box; it was levelled off as before. The weight of the grains around the loaf and the volume of grain displaced by the loaf were calculated using the following formula:

Seeds displaced by loaf (L) = C (g) + overspill weight – weight of seeds (g), where C is the mean value for seed weight (Equation 1)

$$\text{Volume}\text{of}\text{loaf}\left(\text{V}\right)=\frac{\left(\text{Seeds}\text{displaced}\text{by}\text{loaf(L)*Volume}\text{of}\text{bucket}\text{or}\text{box}\right)}{\text{Weight}\text{of}\text{seeds}}$$ Equation 2

## Calculation of bread nutrient composition

The nutrient composition of bread was determined by calculating yield factors (YFs) and retention factors (RFs) as stipulated by the European Food Information Resource (16). The nutrient levels in the dish were calculated by first finding the values from the United States Department of Agriculture (USDA) food composition tables (17).

Yield factors were calculated using the following formula:

$$\text{YF}=\frac{\text{prepared}\text{dish,}\text{edible}\text{part}(g)}{\text{Total quantity of ingredients }(\text{ready}-\text{to}-\text{cook})\;(\text{g})}$$ Equation 3

Retention factors were calculated to find the amount of nutrients retained after preparation (18). Weight YFs were included in the experimental determination of the nutrient RFs, and the following formula was used:

$$\text{RF}=\frac{\text{Total quantity of ingredients }(\text{ready}-\text{to}-\text{cook})(\text{g})}{\text{Total quantity of ingredients }(\text{ready}-\text{to}-\text{cook})(\text{g})}*\text{YF}$$ Equation 4

## Data analysis

Data entry and data analysis were done using SPSS version 20. Two-way analysis of variance (ANOVA) was used to generate the means and determine levels of significance for the results. Principal component analysis (PCA) was performed to determine sensory attributes of importance in grain amaranth–wheat bread.

## Results

### Comparison of choice of bread samples based on gender

Six samples of bread improved with ascorbic acid were given to each of the male and female testers. Data presented in Table 1 show results of how the testers made their choices. The testers showed no significant difference (*p* < 0.05) in their choice of the bread samples.

**Table 1:** Effect of gender on choice of the bread samples improved with ascorbic acid, fungal α-amylase and a combination of the two with different levels of grain amaranth flour

Improver	Gender	0%	5%	10%	15%	20%	25%	Sig.
AA	Male	7	8	8	9	6	3	0.38
	Female	11	11	9	4	3	1	
AFA	Male	10	5	2	14	4	6	0.90
	Female	12	6	1	12	4	4	
FA	Male	12	5	17	1	3	3	0.99
	Female	12	4	18	2	2	1	

AA, ascorbic acid; AFA, combination of ascorbic acid and fungal α-amylase; FA, fungal α-amylase.

## Recipe standardisation

After several trials of baking and random preliminary sensory evaluation done by students and staff members, sugar was reduced to 30 g based on the recommendations received, while the rest of the ingredients remained the same. The initial 60 g of sugar as indicated in the adopted method by Tang et al. (8) made the breads sweeter than what the random testers are used to eating. The use of butter presented operational challenges; therefore, it was replaced with the same amount (32 g) of vegetable oil.

Fermentation times were also altered from 15 to 20 min for the first fermentation to improve dough leavening. The second fermentation was reduced from 90 to 45 min because it was observed that the dough that was fermented for 90 min was too sloppy and did not achieve oven spring once put in the oven. This could be attributed to the addition of AA and FA that provided optimal acidic conditions and degraded molecules for fermentation by yeast to occur faster. This led to the yeast being overspent; therefore, it was unable to continue to ferment in the first few minutes the dough was placed in the oven (19), resulting in the production of flat bread. Therefore, the times were changed to still have yeast working in the oven to produce a good bread shape.

The advantage of baking grain amaranth–wheat blended bread is that it does not require sophisticated technology. The use of makeshift ovens with controlled heating is enough to produce high-quality bread that consumers can equally enjoy.

### Consumer acceptability

In this study, consumer acceptability tests were used to assess the appearance, aroma, taste at first bite, graininess, aftertaste and overall acceptability of the test breads. The results indicated that there were significant differences (*p* < 0.05) for all bread samples on all the attributes tested for breads improved with ascorbic acid (AA) (Table 2).

**Table 2:** Degree of liking of grain amaranth–wheat flour breads with various grain amaranth flour per cent compositions improved with ascorbic acid

Sample	Appearance	Smell/Aroma	Taste at first bite	Roughness	Aftertaste	Overall acceptability
0AA	2.21 ± 1.33^a^	2.41 ± 1.30^b^	1.48 ± 0.54^a^	3.01 ± 2.03^ab^	2.52 ± 1.56^b^	2.84 ± 1.84^ab^
5AA	2.09 ± 1.18^a^	2.16 ± 1.37^a^	2.26 ± 1.45^ab^	2.32 ± 1.50^b^	2.29 ± 1.47^b^	2.43 ± 1.49^b^
10AA	2.43 ± 1.20^a^	2.00 ± 1.65^a^	2.64 ± 1.70^b^	3.65 ± 2.03^a^	3.60 ± 2.16^a^	2.82 ± 1.60^ab^
15AA	1.59 ± 0.66^b^	2.63 ± 1.55^b^	2.91 ± 1.51^c^	2.33 ± 1.30^b^	2.63 ± 1.64^b^	2.96 ± 1.75^ab^
20AA	2.60 ± 1.65^a^	3.30 ± 2.41^c^	3.55 ± 1.23^c^	3.72 ± 2.17^a^	3.66 ± 2.17^a^	3.48 ± 2.12^a^
25AA	2.70 ± 1.82^a^	2.83 ± 1.63^bc^	2.86 ± 1.63^cc^	4.01 ± 2.34^a^	3.74 ± 2.36^a^	3.59 ± 2.21^a^
*p*-value	0.000	0.000	0.000	0.000	0.000	0.001

Values in the table are means ± standard deviations.

Means with different superscript in the same column were significantly different (*p* < 0.05).

Hedonic Rating Scale used; 1 = like extremely to 9 = dislike extremely.

AA, ascorbic acid.

**Fig. 4:**
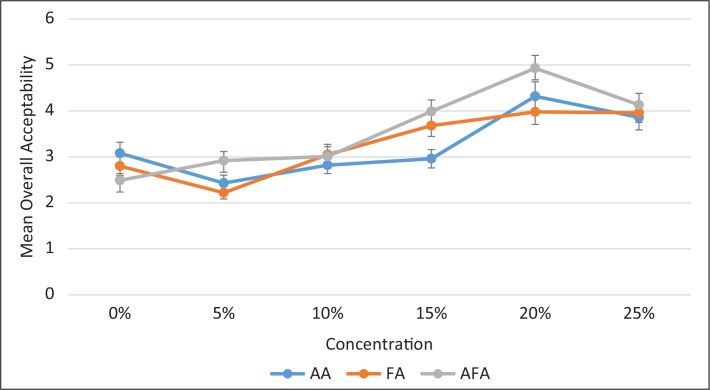
Comparisons of the overall acceptability of the three types of bread samples improved with ascorbic acid, fungal α-amylase and a combination of the two at different per cent compositions of grain amaranth flour.

AA, Ascorbic acid; FA, fungal α-amylase; AFA, combination of ascorbic acid and fungal α-amylase.

Similar trends were observed for bread samples improved with FA and the combination of ascorbic acid and fungal α-amylase (AFA). Overall acceptability showed that ascorbic acid performed better for 10 and 15%, while FA gave better results for 5 and 20% formulations. The combination of the two worked better for the control bread, although it was not very different with the others at 25% blending (Fig. 4).

### Preference ranking test

**Fig. 5:**
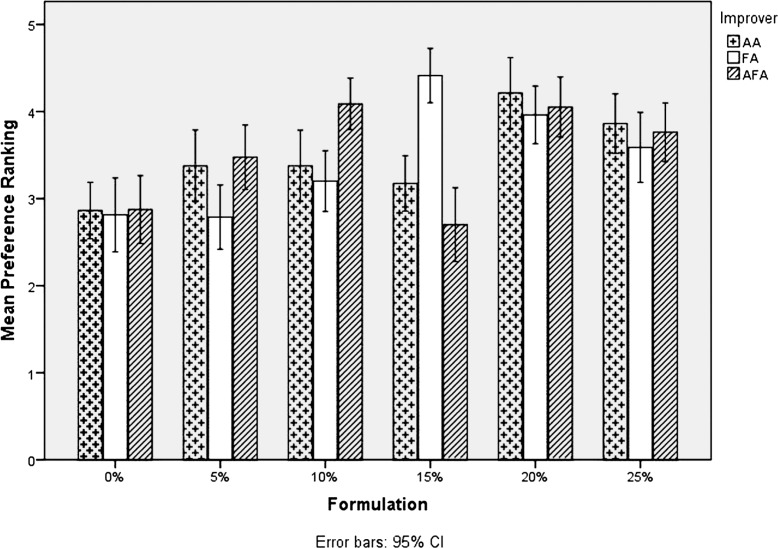
Comparison of preference rankings for different breads.

Preference ranking test showed that consumers preferred 15AFA, 5FA and bread baked with 100% wheat flour regardless of the improvers used. Despite better mean scores for 5 and 10% breads in overall consumer acceptability, 5% grain amaranth bread improved with FA and 15% bread improved with AFA were the most preferred of the test breads (Fig. 5).

### Results for descriptive test

A total of 17 descriptors were generated to describe the appearance, taste, aroma and texture of the breads. These descriptors included brownness of upper crust, brownness of lower crust, crumb colour, alveolus size, alveolus regularity, overall aroma, earthy smell, yeasty smell, saltiness, sweetness, aftertaste, chewiness, crust crispiness, crumb moistness, elasticity, roughness or graininess, and stickiness. Out of the seventeen descriptors mentioned above, nine showed significant differences (*p* < 0.05). PCA indicated the ability of the panel to discriminate the bread samples as presumed.

### Bread improved with ascorbic acid and fungal alpha amylase

**Fig. 6:**
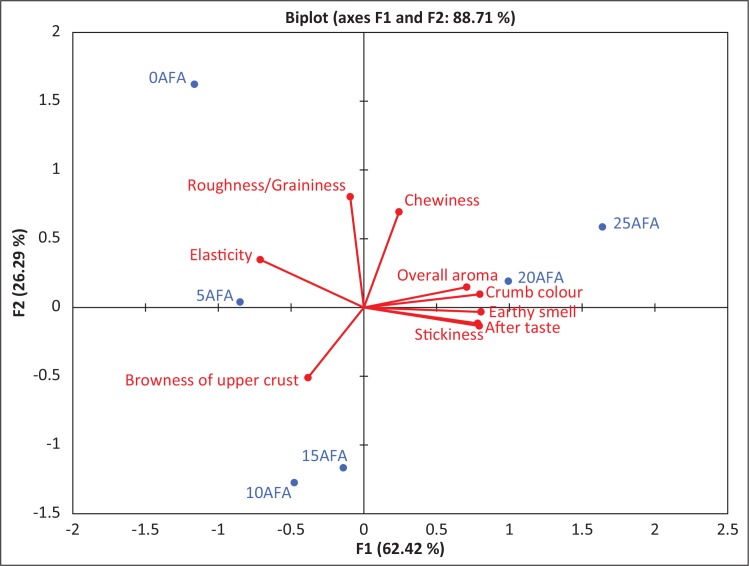
Projection of the bread samples 0AFA, 5AFA, 10AFA, 15AFA, 20AFA and 25AFA on PCA plots. AFA, Bread samples improved with a combination of ascorbic acid and fungal α-amylase.

Attributes that were significantly different were further analysed by the PCA. The results obtained showed that the panel described 88.71% of the variation and perceived 5AFA and 10AFA, 20AFA and 25AFA to have similarities in the tested attributes based on their proximity with each other in the map. It perceived the control bread to be totally different from the rest of the test breads (Fig. 6).

### Physical characteristics of breads

**Fig. 7:**
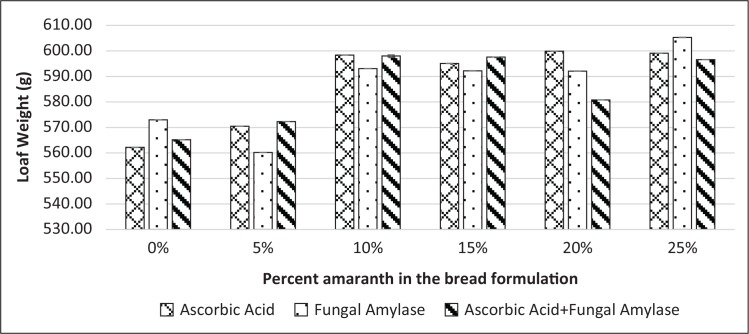
Average weight of bread samples (0, 5, 10, 15, 20 and 25% amaranth) improved with ascorbic acid, fungal α-amylase and a combination of the two.

Average weight after cooling indicated that there was an increase in weight with increased amaranth substitutions in all the test breads (Fig. 7). Oven spring and loaf showed significant differences (*p* < 0.05); they were higher in breads that had lower levels of grain amaranth flour (Table 3).

**Table 3:** Loaf volume and oven spring for bread samples for all treatments

Attribute	Treatment	Bread samples						

		0%	5%	10%	15%	20%	25%	*p*
Loaf volume	AA	2709.47 ± 4.47^b^	2691.20 ± 3.20^c^	2721.65 ± 3.65^a^	2520.72 ± 3.72^f^	2569.43 ± 4.43^e^	2605.96 ± 0.04^d^	0.00
	FA	2879.95 ± 3.95^a^	2825.16 ± 4.16^b^	2752.09 ± 2.09^c^	2752.09 ± 1.09^c^	2538.99 ± 2.99^e^	2605.96 ± 2.96^d^	0.00
	AFA	3275.72 ± 4.72^a^	2849.51 ± 0.51^b^	2831.24 ± 3.24^c^	2691.20 ± 3.20^d^	2557.25 ± 0.25^e^	2685.12 ± 2.12^d^	0.00
Oven spring	AA	3.27 ± 0.12^a^	3.23 ± 0.12^a^	3.13 ± 0.15^a^	2.67 ± 0.12^bc^	2.30 ± 0.17^b^	2.37 ± 0.15^c^	0.00
	FA	3.37 ± 0.15^a^	3.30 ± 0.26^ab^	3.10 ± 0.10^ab^	3.03 ± 0.15b	2.43 ± 0.21^c^	2.37 ± 0.06^c^	0.00
	AFA	3.53 ± 0.15^a^	3.50 ± 0.20^a^	3.23 ± 0.12^b^	3.17 ± 0.12^b^	2.67 ± 0.06^c^	2.53 ± 0.12^c^	0.00

Values in the table are means ± standard deviation.

Means with different superscript in the same column are significantly different (*p* < 0.05).

AA, bread improved with ascorbic acid; FA, bread improved with fungal α-amylase; AFA, bread improved with a combination of ascorbic acid and fungal α-amylase.

### Nutrient composition

Table 4 shows the calculated nutrient composition of the breads. Calculations used 0.90 and 1 as yield and retention factors (18), respectively. The results were based on the recipe that was used throughout the study. The varying nutrient contents of the blended bread were an indication that addition of grain amaranth flour assisted in increasing the nutrient content of the breads. Carbohydrate content decreased with increased grain amaranth additions. Micronutrients were only available in very small amounts.

**Table 4:** Calculated results for nutrient contents of wheat–grain amaranth bread

Nutrient	Amaranth in bread flours						GA
	
	5%	10%	15%	20%	25%	0%	100%
Protein (g) Lysine g/16gN	6.65 7.95	6.76 8.73	6.86 9.37	6.96 10.00	7.06 10.64	6.55 7.46	13.56 5.95
Fat (g)	6.19	6.37	6.544	6.72	6.89	6.02	7.02
Carbohydrate (g)	46.06	45.81	45.56	45.31	45.07	46.31	65.25
Fe (µg)	3.0	3.0	3.3	3.4	3.5	3.1	7,600
Zn (µg)	0.75	0.81	0.87	0.92	0.98	0.70	2,900
Ca (mg)	0.01	0.02	0.02	0.03	0.03	0.00	159
Mg (mg)	0.03	0.04	0.04	0.05	0.06	0.02	248

Source: Dokok et al. (19)

Values for lysine: 5.95g/16gN and 2.90g/16gN for grain amaranth and wheat flours, respectively.

GA, 100g grain amaranth flour.

### Discussion

Wheat flour used in Malawi for bread production is imported from South Africa. Hence, the importation and associated production costs make bread expensive, especially to the rural masses in the country the majority of whom live on less than US$1a day. There is a huge need to use locally available raw food stuffs in the bakery of bread to increase the variety and utilisation. The present study modified wheat bread by adding grain amaranth flour as an ingredient. Amaranth grows wildly in rural area and is culturally eaten as relish for different staples. Paradoxically, the amaranth grain which is equally nutritious is not edible in any form and therefore neglected. The findings of this study can help provide valuable information for consumers, health care practitioners, the government and humanitarian organisations working to improve nutrition in the country.

One key finding of the study was that there were no significant differences in the choice of breads by male and female testers (Table 1). Even though taste sensitivity differs between males and females in real-life situations (20), our results showed that the choice of bread samples was not gender dependent. Analysis of descriptive data from sensory tests showed that testers accepted and preferred bread with 5–15% grain amaranth flour. Acceptability of the test breads decreased as the amaranth flour in the bread increased. The dark colour of the breads, which is typical of grain amaranth, was mild at the aforesaid concentrations compared to when 20–25% additions were used. For instance, testers could easily detect sensory deviations from the common wheat bread when the latter substitutions were added. Control bread had the highest mean score for taste at first bite, which can be attributed to the taste of wheat flour bread that the testers were used to. Lower scores with higher amaranth levels were due to the grain amaranth flour interfering with the actual taste of wheat flour bread. The aftertaste of the bread samples increased in breads with high levels of grain amaranth flour, attributed to the slight bitter taste of grain amaranth (7).

The study used AA and FA enzyme as improvers to enhance the acceptability of the test breads. It hypothesised that breads with up to 25% grain amaranth flour can enhance the utilisation of the grain and be equally preferred by the testers. However, based on the results presented, even with improvers, the addition of amaranth flour as an ingredient to bread and probably other foods should not exceed 15%. The use of improvers helped raise 5, 10 and 15% breads close to the control, while 20 and 25% formulations had reduced oven spring and loaf volume. The explanation for this result is that the improvers degraded complex molecules in 5, 10 and 15% blends, but in the 20 and 25% flours combinations insufficient material was produced for gas production by yeast. Consumers are used to eating bread that is more porous, meaning that higher amounts of grain amaranth flour in bread will not be favoured at all.

Table 4 shows the nutrient composition of the test breads. The addition of amaranth flour contributed protein, in particular, lysine – an essential amino acid necessary for human health. There were also slight increases in iron, zinc and magnesium. Frequent consumption of the test breads, as a staple or co-staple, would assist in curbing micronutrient deficiencies that are of public health concern in Malawi and other developing countries. Taken together, using grain amaranth flour in bread is cheaper than commercial powders and enhancers currently used by the food industry. The increase in the micronutrients as grain amaranth flour is increased can also help commercialise and raise popularity of this ethnic crop (21).

In Malawi, the government and humanitarian organisations promote production of dark green leafy vegetables in home gardens as one way of addressing micronutrient deficiencies among women and under-five children. Grain amaranth could be successfully used in cookery of different foods besides bread to make them more nutritious.

Investigations on the value of adding grain amaranth have remained scanty in Malawi and other countries in Africa. For example, in Malawi, only Gonani (6) has developed bread from amaranth and the work remains unpublished. The other study (7) conducted elsewhere developed biscuits. Nevertheless, both studies reported results similar to those found in the present study. Bread and biscuits with higher amounts of grain amaranth flour were darker in colour and less acceptable by the study subjects, confirming the need to use grain amaranth in lower amounts in foods to enhance acceptability. Given the disparities in cookery and consumer preferences and in light of the paucity of studies undertaken to date, there is a need for more research in the vast majority of countries to understand better the physiochemical, antioxidant and functional properties of the grain.

More so, physical characteristics of breads indicated that values for average weight (Fig. 7) for the control bread and that substituted with 5% grain amaranth flour were close to each other. The compact network in bread dough with higher amounts of grain amaranth flour resulted in less evaporation of the water during baking, leading to heavy bread. However, the addition of AA and FA improved the volume of the breads since they aid in gas production (22). Loaf volume is related with oven spring. When oven spring is higher, the volume of bread is also higher.

The study had some limitations. The rising of the breads was affected as grain amaranth flour substitutions increased due to reduced effect of gluten. During sensory evaluation, no carrier (e.g. tea and bread spread) was used. Notwithstanding these limitations, the study concludes that grain amaranth can be used in breads and examined in other products to enhance diversity and nutrition.
